# Crafting During Coronavirus: participatory methods with older adults during COVID-19

**DOI:** 10.1007/s00391-023-02174-3

**Published:** 2023-04-24

**Authors:** Naomi Clarke

**Affiliations:** School for Policy Studies, University of Bristol, BS8 1TZ Bristol, UK

**Keywords:** Research, Creative, Digital, Participation, Older adults, Forschung, Kreative Methoden, Digitale Integration, Partizipation, Ältere Erwachsene

## Abstract

When coronavirus disease 2019 (COVID-19) took hold, the everyday voices of older adults were frequently overlooked politically, socially and economically. The Crafting During Coronavirus creative, participatory project sought to explore the everyday role of crafting during COVID-19. It was imperative to conduct research with participants in a way that could be a source of positivity especially during a crisis. Using a combination of digital and tangible methods (diaries, photographs, emails and crafted postcards), this project aimed to create a space where participants could narrate, shape, revisit and reflect upon their experiences and their making at a time that suited them. Combining these approaches in a flexible manner, allowed data to be collected and pieced together with older participants to form a patchwork with breadth and depth of everyday narratives during COVID-19.

## Abstract (de)

Als COVID-19 um sich griff, wurden die Stimmen der älteren Bevölkerung in politischer, sozialer und wirtschaftlicher Hinsicht häufig überhört. Das kreative, partizipatorische Projekt „Crafting during Coronavirus“ wollte die alltägliche Rolle verschiedener Gestaltungsmöglichkeiten während der COVID-19-Pandemie untersuchen. Dieser Forschungsansatz sollte in Krisenzeiten eine Quelle der Positivität für die Teilnehmenden darstellen. Mit einer Kombination aus digitalen und greifbaren Methoden (Tagebücher, Fotos, E‑Mails und selbstgestaltete Postkarten) sollte ein Raum geschaffen werden, in dem die Teilnehmenden ihre Erfahrungen und ihr Schaffen zu einem für sie geeigneten Zeitpunkt erzählen, gestalten, überdenken und reflektieren konnten. Die flexible Kombination dieser Ansätze ermöglichte es, während der COVID-19-Pandemie Daten zu sammeln und gemeinsam mit den älteren Teilnehmenden zu einem Patchwork aus alltäglichen Erzählungen zusammenzusetzen, das eine große Bandbreite und Tiefe aufweist.

## Introduction

When coronavirus disease 2019 (COVID-19) took hold, the everyday voices of older adults were frequently forgotten about politically, socially and economically. People aged 65+ years were often grouped as a single at risk, homogeneous group with public policy messaging cementing ageist framing of older adults as frail and vulnerable [6, 11, 20]. Such positionings oversimplify the lived experiences of older adults thus ignoring the heterogeneous experiences of individuals during the pandemic as well as the adaptability, ability and autonomy of older adults [6, 20].

Despite a multitude of texts exploring creative research approaches [7, 14, 16], few have offered detailed methodological explorations of the in-practice use of creative and digital participatory research with older people during COVID-19. Consequently, this article focuses on showcasing the role that digital technology plays in participatory, person-centred research with older people in a way that could be inclusive and positive for all involved. This article aims to offer:

- A combination of theoretical and reflexive positionings on creative and digital research with older adults during COVID-19.
- Discussions and reflections on the use of digital, diary and visual methods in creative research with older adults during COVID-19.

Whilst Derrer-Merk et al. [6, p. 902] state that “extraordinary situations call for extraordinary research”, this article aims to show that utilising creative and flexible person-centred approaches within participatory research with older people need not be seen as extraordinary at all; in fact such approaches should be seen as a necessity in conducting methodologically and ethically sound participatory research.

## Theorising craft, researching creatively and gerontological research

Informed by social constructionist and feminist approaches disrupting traditional patriarchal “knowledge” positionings, this research builds upon the assertion that knowledge is not a static finality, but a social construct affected by external influences [9, 25]. Materials and things are vital, entangled elements of our social world that co-create how we relate to others, things, and understandings [27]. Concurrently, this research is shaped by sociomaterial and material gerontological approaches that move away from a biomedical view of ageing as a biological process. Material gerontological approaches view ageing as a complex entanglement of humans, materials, spaces and the relationships and interactions between them [15]. As such, there is potential for the outcomes of material gerontological research to be more responsive to these complex entanglements of lived experiences, older people, digital technologies and materials [15].

The Crafting During Coronavirus (CDC) research utilised a very broad, initial research question inviting older people to narrate their own experiences of using craft during crisis in a way that was flexible and open-ended: “What are the contextually situated lived experiences of crafters and craft during COVID-19?”

## Study

### Participant group: size and recruitment

Selecting participants should be done in a way that allows for deep exploration of the research question [1]. Given the broad nature of the research, and the fact that larger participant groups can be beneficial for offering diversity within the topic, I did not set a limit on participant numbers. As well as being a PhD student, I am also a crafter with 25 years experience of making. This dual role enabled me to utilise my creative background as well as legitimise my position as a crafter and a researcher. Following ethical approval from the University of Bristol, I shared the research project on multiple social media platforms (Facebook, Twitter, Instagram) with a direct weblink to my website for project information, ethics and consent forms [2].

After sharing the digital call-out for participants, 317 people actively participated in the Crafting During Coronavirus (CDC) research. The average age of participants was 53 years (ages ranged from 21–84 years), a majority (229) were UK based (although participants were in 19 countries in total) and female (313 female, 1 male and 3 identified as non-gender binary). There were 28 participants aged 70–84 years and located in 5 countries (Australia, Canada, Slovenia, UK, USA).

Once I had received the digital consent form, I emailed participants individually to introduce myself, share a digital diary “template” with information about possible diary headings (date, location, what they are crafting, if they are using social media, feelings, any other thoughts, and photographs of their craft) and encouraged them to use the template flexibly so that it would work for them. I invitied participants to keep a diary whenever they crafted (this could be by hand, digitally, through stitch or another form) and to send it back to me via email (either written in the email body, or as an attachment) along with photos of their making (again, either in the email body or as an attachment).

Using digital connections is complex; whilst it enables broader geographical connections with participants than face-to-face methods, it also limits participation to those with digital access and knowledge [19]. I am therefore aware that those who took part in the CDC research are likely to be people who already have some element of digital access and knowledge. During COVID-19, however, face-to-face research approaches were simply not an option for both researcher and participant safety. Consequently, digital approaches had to be adopted within this research.

It is the experiences of these older 28 participants who have shaped the writing of this article to which I now turn to in more detail. These experiences are explored through the lens of material gerontology, so as to look relationally at what people, things and technologies do and become within the research process and how they respond to one another and themselves [15].

## Methods

I have chosen to examine this relational entanglement of things, people and technologies through the lens of three method modalities that were utilised within the Crafting During Coronavirus research:

1. Digital methods with older adults.
2. Diary methods with older adults.
3. Visual methods with older adults.

I aim to make visible assumptions that are often taken for granted within ageing and technological practices as well as creative research approaches.

### “Crafting During Coronavirus”: Digital methods with older adults

During the COVID-19 pandemic, when there was a global “stay home” message to minimise the spread, digital technology became a necessity for communications, engaging in leisure activities, work, and accessing services [22]. Far from being reluctant or unable to use digital technology, many older adults were active everyday users of digital technology [22]. Digital inequalities, however, exist amongst older adults actively online and viewing digital inequalities as binary between those with internet and those without is too simplistic and only tells part of the story [8]. Older adults are a diverse group and an individual’s digital technology use is influenced by multiple psychosocial factors including prior experiences in employment, social activities, knowledge, education and motivations as well as comfort and confidence in technology [4, 8].

This complex entanglement of experiences, technologies and older people was clear in the use of digital methods in this project. I wanted the digital diary to be flexible, so the individual participant’s agentic position was centred. For some, this flexibility meant combining digital technology with more tactile, tangible methods that they felt confident using:

“As I don’t have Word I find that getting my diary & photos to you bit awkward. However think I have been able to give you what you want by using Notes on my I pad. Although photos don’t always seem to send. I have printed and kept all my diary entries plus photos and put together. This will be available for your use.” Josephine, 77, UK

Far from being put off or reluctant to use digital technology, it was evident that older people have great creativity in using, adapting and combining digital technology with other activities in order to make it work for them in a way that is beneficial.

This flexibility and adaptability created space for the older people to not only reflect on how they wanted to keep a digital diary but to also reflect on the role of craft, time, and self:

“Once I had settled on the presentation of the diary in a journal form I decided that taking photos of the items made with perhaps the inspiration behind them was a simple enough task. Some of the projects often took longer than others so there would be a time overlap but I have certainly been surprised how much time I spend crafting normally. It’s having the time to think and create that I’m enjoying.” Lily, 71, UK

Using digital technology with older people was not without difficulties though. Whilst I tried to regularly check in with participants, there were times that I missed opportunities to support interactions with digital technology:

“I wish I didn’t have to go into Naomi’s webpage every time to find the diary template. I perhaps don’t, but am not very tech savvy. It would be good to have a BLANK diary form that I can just keep in Word and copy and use it each time.” Susan, 70, UK

When using digital methods, it is important to consider how easy a device or digital method is to use. If digital technology is difficult to use, this can lead to the older adult questioning their own ability and self-worth as well as their ongoing engagement with digital technologies [26]. Where digital technologies are useful and usable, they are more likely to be beneficial for older people [22]. This fine line of balancing the entanglements of usefulness, usability and digitality for older people was clear:

“I love seeing other people’s creations but it can also affect my own confidence. I am a bit of a technophobe and although I can manage basics … my children all wanted to zoom call on Sunday … lovely to see them but I had no sound!” Theresa, 70, UK

There were times, therefore, that technology offered both connections and frustrations for older people, not just in this research but in day-to-day pandemic life.

Social and digital inclusion, both in this study and in everyday pandemic life, is more than simply providing digital access. There *is* the potential for digital methods to support social connectedness. Digitality enables potential connections with millions of others in ways like never before [7]. It enables us to build new, virtual communities and to create, recreate and redefine our identity [24]:

“I have also realised that I tend to be a solitary crafter—I don’t belong to quilting or sewing groups. Mostly this is because I have felt inhibited by my (perceived) lack of skill. Engaging with groups on Facebook, however, has made me realise that I am a better sewer than I thought! I think the relative anonymity has helped me share my work.” Abigail, 71, Australia

When we were told to physically distance during COVID-19, many older adults were simply labelled as “at risk” which cemented stereotypical, ageist messaging [6, 11, 20]. In doing so, the social connectedness of many older adults grew smaller:

“I am in the demographic that is at risk, being 70 and with a pre-existing heart condition, so only go out to walk and to local shops for groceries. Staying at home suits me, though I miss my friends and interest groups that I am part of. Therefore, having specific tasks suits me.” Michelle, 70, Australia

Whilst not a substitute for in-person interactions and tactility, technology offers some solace in that we can be on our own yet never be alone [24]:

“It connects me directly to you (even if at times by an automatic response), and indirectly with other crafters involved, and I feel less lonesome.” Ladka, 70, Slovenia

Digital connections therefore became increasingly important during COVID-19 [5] but so too did the *quality* of these digital connections. Personal relationships carry a high importance and can be supported and maintained through digital technologies [26]. This complex entanglement of relationships, digital technologies and materials was evident not only in how older people responded to the digital methods in the CDC project but also in their daily pandemic experiences. The older people were not reluctant to use digital methods; they were incredibly creative in how they adapted their use to make the research project work for them too.

### “Crafting During Coronavirus”: Diary methods with older adults

Narratives permeate every aspect of our world allowing us to connect with others and transcending generational and geographical constraints. Narratives enable us to define who we are and who we are not [21]. Open approaches to diaries, where there is a set of instructions at the start of the diary and the participant can then structure the diary thereafter, work well within narrative research as the participant has more freedom over how they construct and narrate their experiences; the agency of the participant is thus centred [17].

Using open diaries can be a key method in research with older adults as it is person-centred allowing individuals to capture and narrate their own understandings and experiences without presuppositions [18]. This plurality, affording people the space to use their own words without the presupposed boundaries and stereotypical homogenising of older people’s voices, is in line with sociomaterial gerontological and person-centred approaches. We were all navigating vast changes within everyday life during COVID-19 when much of our world was made smaller and felt increasingly out of our control:

“The day before I made this I went to our nursery, which required an appointment, only the second time out since 3/4 … my husband picks up the groceries at curbside. I almost had a meltdown because the feeling of claustrophobia from negotiating my mask, my hearing aides, my glasses and gloves. So many new rules.” Patricia, 79, USA

Given the changes we were all dealing with, it felt ethically imperative to use methods that did not add in new tasks that could felt overwhelming or onerous:

“As a writer, jotting down a few words each day is neither a problem nor a burden. I was intending to do this anyway in order to have a record of what are, by anybody’s reckoning, the most extraordinary times of our lifetimes. This project has given me the incentive to do that and to do it on a regular basis.” Evelyn, 70, UK

It is also possible, however, that some participants may find the lack of structure anxiety provoking. Meth [17] suggests this anxiety could be minimised through the researcher “checking in” with participants to see how they are. On an ethical, and person-centred basis, I frequently “checked in” with each participant to see how they were, how they were finding the diary process and if there were any changes that could help. I encouraged participants to use the diary structure as a guide, but I also emphasised that it is flexible and can be adapted to suit that individual:

“I am a tentative blogger so this serves a double purpose, as technology is still tricky for me. Thank you for giving me the opportunity of sharing, and your patience with my attempts. The crafting is an added bonus of pleasure.” Michelle, 70, Australia

I encouraged participants to play with diary modality to make it work for them. The method of diary-keeping varied with participants utilising various approaches including handwriting, typing it in the body of an email, mixed media (paint, pens, sewing), photos and more. Whilst this could be seen as problematic (how do you store and analyse^1^across creative data forms?), I felt that it was advantageous within this project as the flexibility and adaptability of the diaries and methods created space for participants to share their narratives at times that work for them, and in ways that allowed them to reflect, build upon, revise and expand on their experiences. As participants did not have to provide an answer on the spot (as they might in a survey or in-person interview), diaries can be used for reflexivity without time constraints [17]. This temporality of diaries can reveal changing understandings, interactions with, relations to and entanglements with others, time, and materials over a prolonged period [18]. This longitudinal perspective can help to make visible that which has typically been taken for granted, which is a key aspect of material gerontological approaches:

“The diaries have helped me to stay on track and work a little more than I might have without being accountable for my work. They have also encouraged me to think about and identify my feelings both about my projects and the quarantine. I am normally not very self-reflective so this is a challenge for me. I have never kept any kind of diary for myself though I tried a few times when I was younger and failed miserably. Your project has given me a task that makes me responsible to someone else.” Sandra, 74, USA

This also provided space for the diaries to act as a springboard for participants to reflect upon other life experiences:

“I am not a good journaller so keeping this diary has provided me with the incentive to reflect on the Covid-19 lockdown experience. I have realised that I craft far more often than I thought. I have also begun to reflect on the importance of craft in my life and that of my family (sisters and daughters) and the legacy of my mother.” Abigail, 71, Australia

Diaries thus offered space for greater self-awareness in relation to one’s own experiences as well as in relation to others, materials, and actions in the present time but also for past reflections and future aspirations.

Using diaries with older adults during a pandemic is complex as it means considering participant confidence, access to materials and digital technologies, literacy, shared language, participant time, agency, ownership and understandings. These multiple factors, however, could also be seen as reflecting the entanglements that are woven throughout sociomaterial gerontology as it means considering this complex interaction between people, things, methods and participation.

### “Crafting During Coronavirus”: Visual methods with older adults

Images do not exist alone; they are subject to continuing interpretation, carry a multitude of meanings, and are deeply embedded within our world [16]. Crafting is also not finite or static; materials have meanings ascribed to them that transform through their relation to other things and people [27]. It was this interwoven relationship between making, makers, and materials that shaped the invitation to older adults to share visuals of their making:

“I love crafting, and this project came at the right time to make some sense of the physical and mental challenges that we have shared as a creative community. You have tapped into a movement that gives sense to the bigger picture in our geopolitical world.” Michelle, 70, Australia

By situating the person as the expert, I welcomed to using visuals to tell their own story. As such, the photos of crafting (in planning, progress, finished) were not data for analysis in themselves, but an elicitation tool and a touchstone object to understand the role of craft in people’s lives:

“At this time of lock down I haven’t been able to share my projects with my like minded friends so sharing the diary has opened up another opportunity for me. This project has also got me into the habit of taking photos of my finished items.” Judy, 73, UK

This taps back into the potential of digital technology for building communities, strengthening relationships, and sharing experiences. It was the use of digital technology, to share the visuals of materials, that created space for a “slow science” where knowledge is generated through reflection and reflexivity [16]:

“I’ve found it really interesting—finding things out about myself! It has made me realise how I operate, how creative I actually am and how impulsive I must be! It seems it might be a historic document in years to come.” Alice, 71, UK

Visual methods with older adults hold potential within participatory research as the power lies with the participant and what they choose to capture within their image [16]. The importance of agency within visual research methods aligns itself with material gerontological approaches that seek to make visible that which has previously gone unseen.

There are, of course, ethics to consider when adopting visual and digital methods. Anonymity and confidentiality are incredibly difficult to ensure within visual research [16, 23]. Disguising images, to offer anonymity, could also be seen to silence participants’ voices [16].

As such, I adopted a flexible approach where I invited participants to *choose* whether to be anonymised both in their craft visuals and their diaries. All too often, the voices of older people have gone unheard, and it was important to offer space for the visibility of the entanglement of craft, materials, digitalities and older people during COVID-19.

## Conclusion

Methods are not simply tools that unlock that which you are studying; methods play crucial and active roles in creating the phenomena being researched [27]. Combining visual, digital and creative approaches within the Crafting During Coronavirus project created space for older adults to share their pandemic craft experiences:

“It is quite true that the diary was a very important part in adjusting to those early pandemic days and the move into the unknown of the first lockdown. For me the diary and the crafting was a valuable focus throughout this uncertain and often frightening time. I often reflect on what fate or circumstance prompted me to respond to your call.” Barbara, 84, UK

Digital spaces have evolved shifting creative practice sites away from specialised sites to broader communities and homes [10]. Creative research approaches support the production of new knowledge individually and collectively which, in turn, challenges traditional, patriarchal, disciplinary norms [10]. It is through increasing older people’s visibility (of their making, lives, experiences, and voices) that we can begin to understand lived experiences.

Stereotypical perceptions of older people being disinterested in digital technologies are oppressive and create barriers [13]. Older people *are* willing to use, and learn to use, digital and online technologies. Older people also have knowledge, experiences, and creativity in adapting such research approaches so that it works for them.

In the context of the Crafting During Coronavirus research project, combining digital, creative, and diary methods allowed data to be collected and pieced together with participants to form a patchwork, with breadth and depth, of everyday narratives during COVID-19 for older people:

“I enjoy being part of your group of crafting correspondents (almost like a secret underground movement), to create a flavour of our disparate life experiences during this often sad and certainly unsettling time in history through our needles and other crafts—perhaps as a form of escapism or hold on reality.” Barbara, 84, UK

A combination of person-centred method modalities supports participatory research with older adults in a way that can challenge traditional hierarchical approaches whilst working with older adults as active agents in the research process to reimagine ageing and participation. This approach thus lends itself to generating new, creative and participatory knowledges and understandings.

Despite the geographical and physical distancing surrounding during COVID-19, there was potential for digital connections to flourish in supporting the sharing of stories for older people. Combining digital, creative, and diary methods holds exciting potential within research with older people. Utilising such methods offers a flexible approach as it is not a binary of empowered vs. exploited, or controlled vs. autonomous [12]. Instead, such approaches sit on a spectrum upon which there are complex interactions as to do the interactions and entanglements between older people, materials and experiences.

## Footnotes

1 The analysis process used in the CDC research is based on Braun and Clarke’s reflexive thematic analysis [1] in combination with using slow-stitch as a creative, material-oriented analytical approach [3].

## Declarations

For this article no studies with human participants or animals were performed by any of the authors. All studies mentioned were in accordance with the ethical standards indicated in each case.
